# An Engineering Prediction Model for Stress Relaxation of Polymer Composites at Multiple Temperatures

**DOI:** 10.3390/polym14030568

**Published:** 2022-01-30

**Authors:** Xiaochang Duan, Hongwei Yuan, Wei Tang, Jingjing He, Xuefei Guan

**Affiliations:** 1 Graduate School of China Academy of Engineering Physics, Beijing 100193, China; duanxiaochang19@gscaep.ac.cn; 2 Institute of Chemical Materials, China Academy of Engineering Physics, Mianyang 621900, China; yuanhw@caep.cn (H.Y.); tangwei@caep.cn (W.T.); 3 School of Reliability and Systems Engineering, Beihang University, Beijing 100191, China

**Keywords:** polymer-matrix composites (PMCs), stress relaxation, analytical modelling

## Abstract

This study develops an engineering prediction model for stress relaxation of polymer composites, allowing the prediction of stress relaxation behaviour under a constant strain, over a range of temperatures. The model is based on the basic assumption that in the stress relaxation process the reversible strain is transformed to irreversible strain continuously. A strain-hardening model is proposed to incorporate nonlinear elastic behaviour, and a creep rate model is used to describe the irreversible deformation in the process. By using stress relaxation data at different temperatures, under different strains, the dependence on temperature and initial strain of the model parameters can be established. The effectiveness of the proposed model is verified and validated using three polymer composite materials. The performance of the model is compared with three commonly used stress relaxation models such as the parallel Maxwell and Prony series models. To ease the use of the proposed model in realistic structural problems, a user subroutine is developed, and the stress relaxation of a plate structure example is demonstrated.

## 1. Introduction

Stress relaxation is a common phenomenon in polymer composite materials and the rate of relaxation can be affected by time, temperature, initial strain, environment, and so on [1,2,3,4]. In the course of long-term service, a pre-stressed part in a structure may lose its prescribed initial stress due to stress relaxation, and structural failure may occur when the retained stressed is less than the threshold value [5,6,7]. Therefore, it is important to accurately predict the time-dependent stress relaxation behaviour for risk mitigation.

In the stress relaxation process, stress decreases under constant strain. The total strain $\varepsilon_{t}$ can be modelled as a superposition of a reversible component $\varepsilon_{r}$ and an irreversible component $\varepsilon_{i}$ such as:

(1) $$\varepsilon_{t}=\varepsilon_{r}+\varepsilon_{i}=\mathrm{const}.$$

The constant strain condition yields a zero variation of the total strain with respect to time, i.e., ${\dot{\varepsilon }}_{t}=0$, and Equation (1) can be rewritten in the form of strain rate as:

(2) $${\dot{\varepsilon }}_{i}=-{\dot{\varepsilon }}_{r}$$

Using the linear viscoelastic assumption, the reversible and irreversible strain rate components in Equation (2) can be expressed as ${\dot{\varepsilon }}_{r}=\dot{\sigma }/E$ and ${\dot{\varepsilon }}_{i}=\sigma /\eta$, respectively, where E is the elasticity modulus and η is the coefficient of viscosity. The integration of Equation (2) over time yields the classical Maxwell stress relaxation model in Equation (3), which describes the relaxation process equivalently using a linear spring and a dashpot connected in series [8]. The parallel Maxwell model [9] and the Prony series model [10] in Equation (4) are two notable variants based on the classical Maxwell model. By adding an additional stretching parameter β (0<β<1) into the exponential term of the Maxwell model, a ‘stretched’ exponential form can be obtained in Equation (3). The ‘stretched’ exponential form was first empirically suggested by Kohlrausch to describe a wide range of slow relaxing in strongly interacting materials and was also postulated by William and Watts for dielectric relaxation, known as the KWW model [11]. It should be noted that apart from the spring-dashpot representation approach, data-driven empirical models are also available in specific applications, e.g., Refs. [12,13,14].

(3) $$\left\{\begin{array}{ll} \sigma \left(t\right)=\sigma_{0}\cdot \exp\left(-\frac{t}{\tau }\right), & \mathrm{Maxwell}\\ \sigma \left(t\right)=\overset{n}{\underset{i=1}{\sum }}\sigma_{i}\cdot \exp\left(-\frac{t}{\tau_{i}}\right), & \mathrm{Parallel}\;\mathrm{Maxwell}\\ \sigma \left(t\right)=\sigma_{\infty }+\overset{n}{\underset{i=1}{\sum }}\sigma_{i}\cdot \exp\left(-\frac{t}{\tau_{i}}\right), & \mathrm{Prony}\;\mathrm{series}\\ \sigma \left(t\right)=\sigma_{0}\cdot \exp\left[-{\left(\frac{t}{\tau }\right)}^{\beta }\right], & \mathrm{Kohlrausch}-\mathrm{Willim}-\mathrm{Watts} \end{array},\right.$$

In Equation (3), σ is relaxation stress, $\sigma_{0}$ is initial relaxation stress, t is time, i is the index of the Maxwell element, n is the total number of the Maxwell elements, and $\tau_{i}=\eta_{i}/E_{i}$ is the Maxwell element relaxation time. The Maxwell model and its variants [15] have been successfully applied to stress relaxation predictions of many polymer composites. For example, barium titanate-silicone elastomer composites [16], glass fibre reinforcements [17], silicon rubbers [18], reclaimed rubber [19], plain weave polymer matrix composites [20], open-cell polymer foams [21], and so on.

In the Maxwell model and its variants, the irreversible strain behaviour is described using a purely viscous damper. By modelling the irreversible strain rate component based on different irreversible deformation mechanisms, the corresponding stress relaxation equations can be obtained. In Ref. [22], the plastic strain rate using Orowan’s equation was adopted for the irreversible component to obtain the stress relaxation model for general crystals. In Ref. [23], the same plastic strain rate model was applied to model the stress relaxation of ultrafine grain aluminium at room temperature. In Ref. [24], the irreversible strain component was further decomposed into the plastic strain and creep strain to model the stress relaxation of titanium alloy at 600 °C. In Ref. [25], the irreversible strain component was modelled as the creep strain with a power law creep rate equation and was applied to describe the stress relaxation of polytetrafluoroethylene composites subject to cylindrical macro indentation. In Ref. [26], the load relaxation behaviour of polymer geogrids was investigated by modelling the irreversible strain component as a non-linear inviscid component and a non-linear viscous component connected in parallel. In Ref. [27], the irreversible part of the standard solid equation as a stress-dependent non-linear inviscid component was used to describe the stress relaxation behaviour of epoxy resins.

In the aforementioned studies, the reversible strain component was usually treated as the linear elastic strain. However, laboratory data show that the nonlinear elastic behaviour is very common in many polymer composites [28,29]. The linear approximation to the nonlinear elasticity can lead to modelling error in predicting the stress relaxation of polymer composites, although such an error can be reduced by increasing the number of Maxwell elements in the parallel Maxwell model and the Prony series model. However, the determination of the required number of Maxwell elements depends on engineering experience and is usually not known a priori for general applications.

In view of this, the goal of this study is to develop a new stress relaxation model by incorporating the nonlinear elastic effect into the stress relaxation process. The novelty of the model is twofold. For one thing, it can reduce the potential linear approximation error in those commonly used models which are based on linear viscoelasticity. For another, the model can eliminate the need for tuning the number of Maxwell elements in existing parallel Maxwell and Prony series models to meet the required fitting accuracy. To this end, a strain-hardening model is adopted, allowing for taking the nonlinear effect into the reversible strain component. A general creep rate model is employed to describe creep deformation. By integration the two components and incorporating the temperature effect into the model parameters, a new stress relaxation prediction model at multiple temperatures is formulated.

The remainder of this study is as follows. First, the detailed construction of the proposed stress relaxation model is presented. Next, the effectiveness of the proposed model is verified using testing data on polymer glass and is subsequently validated using testing data on FM-73 adhesive. The performance the proposed model is further compared with three commonly used stress relaxation models using testing data on HDPE. Following that, a user material subroutine of the proposed model is developed for the ANSYS finite element package, allowing for applying the proposed stress relaxation model to practical engineering problems with parts having irregular shapes. The stress relaxation of a plate-like structure is demonstrated.

## 2. Stress Relaxation Model Development

**Figure 1:**
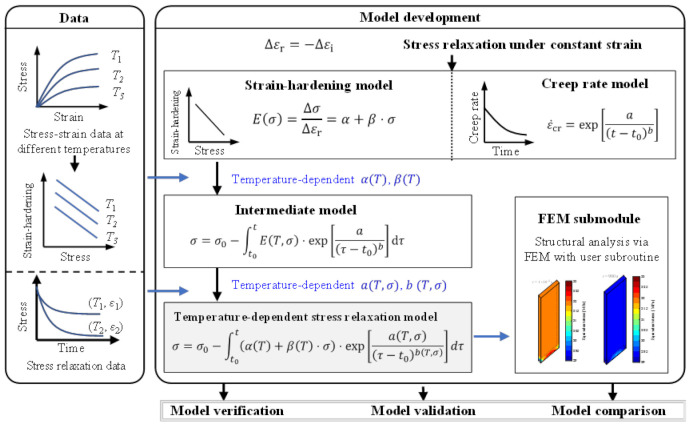
The overall modelling process.

Figure 1 presents the overall process of model development, verification, validation, and comparison. In the part of model development, the hypothesis that stress relaxation is a crossover from reversible deformation to irreversible deformation is used. Under this hypothesis, the stress-dependent strain-hardening model and a creep rate model are employed to model the nonlinear elastic deformation and creep deformation, respectively. The stress–strain testing data at different temperatures are used to estimate the parameters in the strain-hardening model. The temperature effect on those parameters is incorporated by using a polynomial fit. In addition, the creep parameters are obtained using the stress-relaxation data at different temperatures and strains. The effectiveness of the developed model is verified using data on several different polymer composite materials. Validation and comparison are made using independent data and with existing models, respectively.

In this study, the total strain in the stress relaxation process of polymer composite material is assumed to be a superposition of the three main components,

(4) $$\varepsilon_{0}=\varepsilon_{e}+\varepsilon_{p}+\varepsilon_{\mathrm{cr}},$$

where $\varepsilon_{0}$, $\varepsilon_{e}$, $\varepsilon_{p}$, $\varepsilon_{\mathrm{cr}}$ are total strain, elastic strain, plastic strain, and creep strain, respectively. Under a constant total strain, the stress relaxation process involves the transformation of the elastic-plastic deformation to the creep deformation, which can be expressed as Equation (5)

(5) $$\Delta \left(\varepsilon_{e}+\varepsilon_{p}\right)=-\Delta \varepsilon_{\mathrm{cr}}.$$

The nonlinear elastic effect is taken into account using the strain-hardening model and is detailed below.

### 2.1. Strain-Hardening Model for Elastic-Plastic Deformation

**Figure 2:**
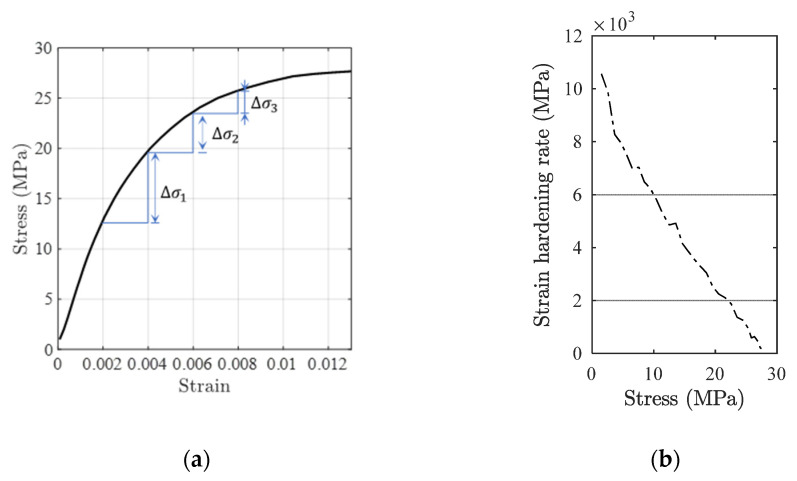
Mechanical behaviour of typical polymer composites. (**a**) Stress–strain curve, and (**b**) strain-hardening rate vs. stress curve.

Figure 2a shows the stress–strain curve of polymer composites under uniaxial compression testing at 20 °C [29], where $\Delta \sigma_{i},\;\left(i=1,\;2,\;3\right)$ are the stress-increment at a continuous fixed increment of strain. It is used here for the purpose of illustration, more detailed information about the material can be referred to [29]. It can be observed that as the loading process, the stress incremental variable decreases ($\Delta \sigma_{1}>\Delta \sigma_{2}>\Delta \sigma_{3}$). Based on the stress–strain curve in Figure 2a, the strain-hardening rate vs. stress results are obtained using Equations (6) and (7), respectively, and the results are shown in Figure 2b for the purpose of illustration.

(6) $$\Theta \left(\sigma \right)=\frac{\Delta \sigma }{\Delta \varepsilon }=\frac{\sigma_{i+1}-\sigma_{i}}{\varepsilon_{i+1}-\varepsilon_{i}},i=1,\;2,\;3\ldots ,n-1,$$

(7) $$\sigma =\frac{\sigma_{i+1}+\sigma_{i}}{2},i=1,\;2,\;3\ldots ,n-1,$$

where $\Theta \left(\sigma \right)$ is the strain-hardening rate, σ is the stress, and ε is the elastic-plastic strain, i.e., $\varepsilon =\varepsilon_{e}+\varepsilon_{p}$. The subscript i is the data point index, and n is the total number of data points.

Based on the results shown in Figure 2b, the following linear strain-hardening model is proposed [30].

(8) $$\Theta \left(\sigma \right)=\frac{\Delta \sigma }{\Delta \varepsilon }=\alpha +\beta \cdot \sigma ,$$

where α and β are fitting parameters. The linear elasticity is recovered when β=0 with α being the regular elasticity modulus.

Knowing that $\Delta \varepsilon =\Delta \left(\varepsilon_{e}+\varepsilon_{p}\right)$ in Equation (8), substitute Equation (8) into Equation (5) to obtain

(9) $$-\frac{\Delta \sigma }{\Delta \varepsilon_{\mathrm{cr}}}=\Theta \left(\sigma \right),$$

or

(10) $$\Delta \sigma =-\Delta \varepsilon_{\mathrm{cr}}\cdot \Theta \left(\sigma \right).$$

For a small time, variation Δt, the following approximation is valid by assuming that $\Theta \left(\sigma \right)$ remains constant in the time increment.

(11) $$\frac{\Delta \sigma }{\Delta t}=-\frac{\Delta \varepsilon_{\mathrm{cr}}}{\Delta t}\cdot \Theta \left(\sigma \right),$$

In continuous form, Equation (11) writes

(12) $$\frac{d\sigma }{dt}=-\frac{d\varepsilon_{\mathrm{cr}}}{dt}\cdot \Theta \left(\sigma \right).$$

It is noted that the actual form of $\Theta \left(\sigma \right)$ in Equation (12) is determined according to the strain-hardening rate vs. stress curve. In this study, the linear equation in Equation (8) is used.

### 2.2. Creep Rate Model for Creep Deformation

A phenomenological full-stage creep rate model previously developed for polymer bonded composite material is adopted in this study [31]. For the stress relaxation process, there is no tertiary creep stage, i.e., the stage approaching creep rupture; therefore, the full-stage creep rate and creep strain models in Ref. [31] are reduced to,

(13) $$\frac{d\varepsilon_{\mathrm{cr}}}{dt}=\exp\left[\frac{a}{{\left(t-t_{0}\right)}^{b}}\right],$$

and

(14) $$\varepsilon_{\mathrm{cr}}=\int_{t_{0}}^{t}\exp\left[\frac{a}{{\left(\tau -t_{0}\right)}^{b}}\right]d\tau ,$$

respectively. In Equations (13) and (14), $\varepsilon_{\mathrm{cr}}$ is creep strain, t is time, $t_{0}$ is initial time, and a, b are fitting parameters. The parameter a is related to the strain rate in the steady-stage stage in creep, and the terms b control the transitions from transient to steady-state stage. More details on the full-stage creep model can be found in Ref. [31] and is omitted here.

### 2.3. Stress Relaxation Model

Substituting Equation (13) into Equation (12), the stress relaxation rate model is obtained as,

(15) $$\frac{d\sigma }{dt}=-\Theta \left(\sigma \right)\cdot \exp\left[\frac{a}{{\left(t-t_{0}\right)}^{b}}\right].$$

Given the initial stress of $\sigma_{0}$, the relaxation stress at a time t can be solved by time integration of Equation (15) from the initial time $t_{0}$ to t as

(16) $$\sigma =\sigma_{0}-\int_{t_{0}}^{t}\Theta \left(\sigma \right)\cdot \exp\left[\frac{a}{{\left(\tau -t_{0}\right)}^{b}}\right]d\tau .$$

In addition, the stress and hardening rate are non-negative in the physical process of stress relaxation, i.e.,

(17) $$\left\{\begin{array}{c} \sigma \left(t\right)>0\\ \Theta \left(\sigma \right)>0 \end{array}.\right.$$

The constraint in Equation (17) is incorporated into Equation (16) using the Heaviside function to obtain the final form of the stress relaxation equation as

(18) $$\sigma =\sigma_{0}-\int_{t_{0}}^{t}\Theta \left(\sigma \right)\cdot \exp\left[\frac{a}{{\left(\tau -t_{0}\right)}^{b}}\right]\cdot H\left[\sigma \right]\cdot H\left[\Theta \left(\sigma \right)\right]d\tau ,$$

where the term $H\left(\cdot \right)$ is the Heaviside function, defined as

(19) $$H\left(x\right)=\left\{\begin{array}{c} 1,x>0\\ 0,x\leq 0 \end{array}\right.,$$

As the strain-hardening rate is independent of creep or other deformation mechanisms, the model parameters in Equation (18) are estimated sequentially. First, the strain-hardening rate vs. stress results are acquired using stress–strain testing data, and the model parameters α and β in Equation (8) are fitted. By using stress–strain testing data at different temperatures, a temperature-dependent model parameter $\alpha \left(T\right)$ and $\beta \left(T\right)$ can be established, as illustrated in Figure 1. Next, the same procedure is applied to obtain the creep model parameters using Equation (18), using the stress relaxation testing data.

## 3. Model Verification, Validation and Comparison

Testing data on three different polymer composites are used for the purpose of verification, validation, and performance comparisons of Equation (18). The sources of the data used are listed in Table 1. The effectiveness of the proposed model is verified based on creep and stress relaxation data of polymer glasses. The proposed model is further validated using independent validation data on FM-73 adhesive. In addition, the proposed model is compared with three existing reference models using High-Density Polyethylene (HDPE) data.

It should be noted that the stress relaxation mechanisms of polymer composites can be divided into physical relaxation caused by macromolecular chain movement and chemical relaxation resulting from the breakdown of covalent bonds [32,33]. In high-temperature, long-time stress relaxation testing, chemical relaxation is the dominant mechanism [34]. However, the testing temperatures for the data listed in Table 1 are all below the glass transition temperature. Therefore, physical relaxation is the dominant mechanism for stress relaxation in this study.

**Table 1:** Source of data used in model verification, validation and comparison.

Material	Data Source		
	Creep Data	Stress Relaxation Data	Stress–Strain Data
Polymer glass	Shen et al. [35]		Tan et al. [36]
HDPE	Kongkitkul et al. [37]		Leshchinsky et al. [38]
FM-73 adhesive	Peretz et al. [39]	Touati et al. [40]	Ishai et al. [41]

To quantify the performance of each model, the residual sum of squares (RSS) error and the root mean square error (RMSE) defined below are used.

(20) $$\mathrm{RSS}=\overset{m}{\underset{i=1}{\sum }}{\left(y_{i}-{\hat{y}}_{i}\right)}^{2},$$

(21) $$\mathrm{RMSE}=\sqrt{\frac{1}{m}\cdot \overset{m}{\underset{i=1}{\sum }}{\left(y_{i}-{\hat{y}}_{i}\right)}^{2}},$$

where $y_{i}\;$ is the actual data, ${\hat{y}}_{i}$ is the prediction data by model, and i=1, 2,…,m represents the index of a total number of m data points.

### 3.1. Model Verification

**Figure 3:**
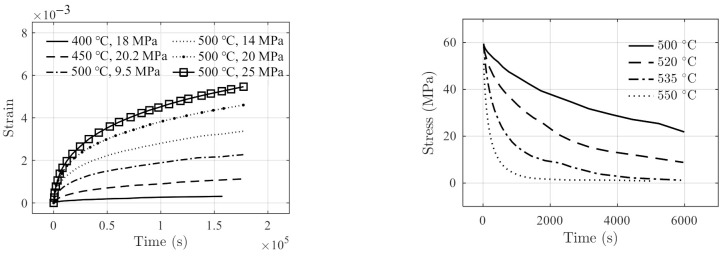
Testing data of polymer glass reported in [35]. (**a**) Creep strain, and (**b**) the stress relaxation.

Data acquired by a 3-point bending test on polymer glass material reported in Ref. [35] are used for model verification. The data include six creep tests and four relaxation tests, as shown in Figure 3.

#### 3.1.1. Strain-Hardening Model Parameters

**Figure 4:**
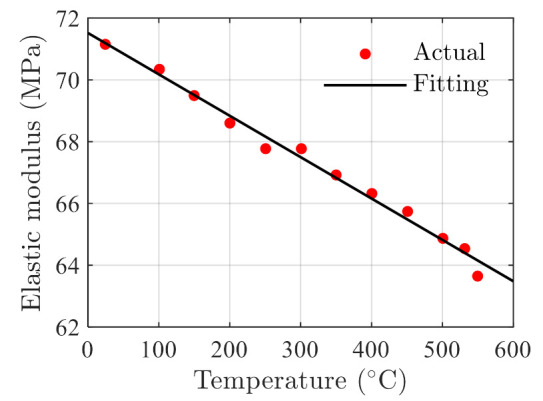
Model fitting results and actual data of elasticity modulus of polymer glass material at different temperatures. The testing data (with legend Actual) are reported in Ref. [35].

According to the stress–strain testing data of polymer glass reported in Ref. [36], the strain-hardening rate is constant for the material, i.e., no strain-hardening for the material. Therefore, the strain-hardening rate, Equation (8), at a specific temperature reduces to

(22) $$\Theta \left(\sigma \right)=\alpha ,$$

where the α is the elasticity modulus. For the hardening rate versus temperature, it can be obtained by fitting the elastic modulus-temperature data in Ref. [35]. The variation of the hardening rate with temperature is shown in Figure 4, where it can be observed that the strain-hardening rate decreases monotonically with the increasing temperature. Therefore, a linear function can be used to describe the relationship between temperature and the strain-hardening rate such as

(23) $$\Theta \left(\sigma ,T\right)=\alpha_{1}+\alpha_{2}\cdot T,$$

where the $\alpha_{1}$ and $\alpha_{2}$ are coefficients. Using the actual elastic modulus data shown in Figure 4, the coefficients are obtained using the least square estimator as

(24) $$\left[\alpha_{1},\alpha_{2}\right]=\left[71.52,-0.01340\right].$$

#### 3.1.2. Creep Model Parameters

The nonlinear least squares estimator is used to obtain the creep parameters for the six sets of creep testing data shown in Figure 3a. The fitting results on parameters $\left(a,b\right)$ are presented in Table 2.

It is shown by the results that the parameters a and b vary linearly with both T and σ; therefore, a first-order response surface model is used to correlate the parameter with variables T and σ as

(25) $$\left\{\begin{array}{c} a\left(T,\sigma \right)=a_{1}\cdot T+a_{2}\cdot \sigma +a_{3}\\ b\left(T,\sigma \right)=b_{1}\cdot T+b_{2}\cdot \sigma +b_{3} \end{array}\right.,$$

where the $a_{i}$ and $b_{i}$ $\left(i=1,\;2,\;3\right)$ are fitting coefficients.

**Figure 5:**
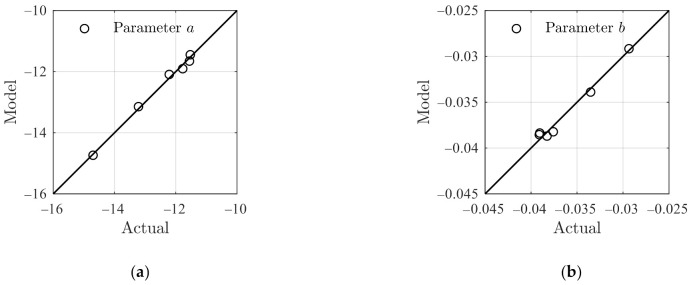
Comparison of the model results on $\left(a,b\right)$ using Equation (25) with that given in Table 2. (**a**) The parameter *a*, and (**b**) the parameter *b*.

Figure 5 presents the comparison between the actual $\left(a,b\right)$ in Table 2 and the model results using Equation (25) where a close agreement is observed, implying that Equation (25) is sufficient to incorporate the temperature and stress effects. The corresponding RSS for the creep parameters $a\left(T,\sigma \right)$ and $b\left(T,\sigma \right)$ are 0.05505 and 1.556 × 10^−6^, respectively.

#### 3.1.3. Stress Relaxation Model

Substituting the temperature-dependent strain-hardening model Equation (23) and creep model Equation (25) into stress relaxation model Equation (18), an intermediate stress relaxation model is obtained as follows.

(26) $$\sigma =\sigma_{0}-\int_{t_{0}}^{t}\Theta \left(\sigma ,T\right)\cdot \exp\left[\frac{a\left(T,\sigma \right)}{{\left(\tau -t_{0}\right)}^{b\left(T,\sigma \right)}}\right]\cdot H\left[\sigma \right]\cdot H\left[\Theta \left(\sigma ,T\right)\right]d\tau ,$$

where the initial relaxation stress is $\sigma_{0}=60$ MPa.

Using the intermediate stress relaxation model Equation (26) to fit the stress relaxation data at different temperatures shown in Figure 3b, the creep parameter coefficients in Equation (25) are obtained by regular least square estimator as

(27) $$\left\{\begin{array}{l} a=\left[a_{1},a_{2},a_{3}\right]=\left[0.08670,0.04548,-54.81\right]\\ b=\left[b_{1},b_{2},b_{3}\right]=\left[-4.109\times 10^{-4},-3.981\times 10^{-4},0.1973\right] \end{array}\right..$$

**Figure 6:**
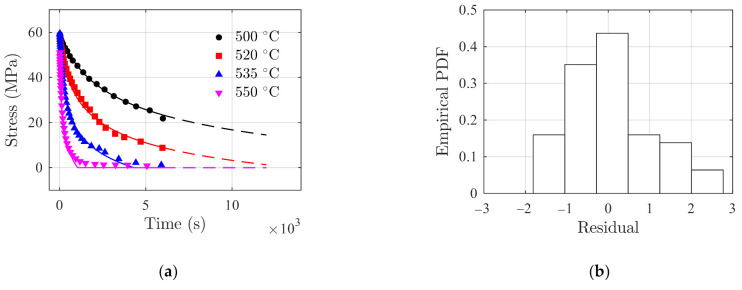
Model prediction of stress relaxation of polymer glass. (**a**) Comparisons of the model prediction results (in lines) with testing data (in discrete markers), and (**b**) histogram of model residuals.

The model prediction results using Equation (26) are compared with the stress relaxation testing data, as shown in Figure 6a. The solid line in Figure 6a shows the fitting results in [0 s, 6000 s], and the dashed line is the prediction results in [6000 s, 12,000 s]. It can be observed that the fitting results are in good agreement with the testing data. The prediction of the stress relaxation at 550 °C reduces to zero and remains zero due to the physical constraint, i.e., the term $H\left[\sigma \left(\tau \right)\right]$ in Equation (26). Figure 6b shows the histogram of model residuals with a standard deviation of 1.002 MPa. It can be observed in Figure 6a that the prediction performance for testing data at 550 °C appears less accurate than the others. This is due to the fact that the fitting parameters are obtained by a global minimization of the sum of the squared error on the data at all testing temperatures as a whole. In this case, the prediction for testing data at 550 °C is less accurate than others.

### 3.2. Model Validation

**Figure 7:**
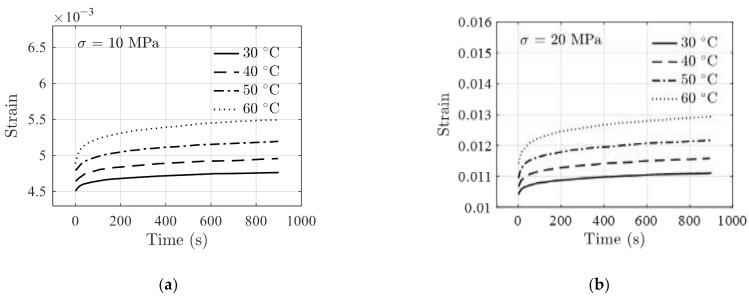
Creep data of FM-73 adhesive reported in Ref. [39] with applied stresses of (**a**) σ=10 MPa, and (**b**) σ=20 MPa.

**Figure 8:**
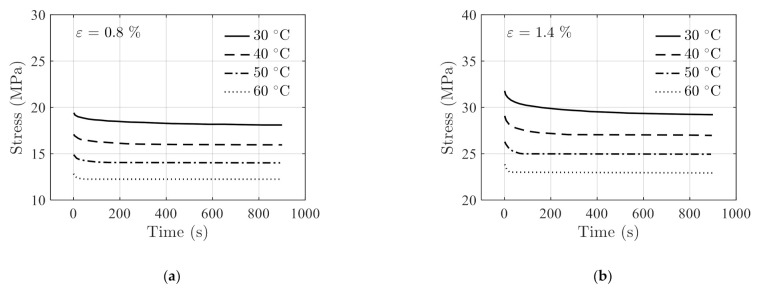
Stress relaxation data of FM-73 adhesive reported in Ref. [40] under constant strains of (**a**) 0.8%, and (**b**) 1.4%.

**Figure 9:**
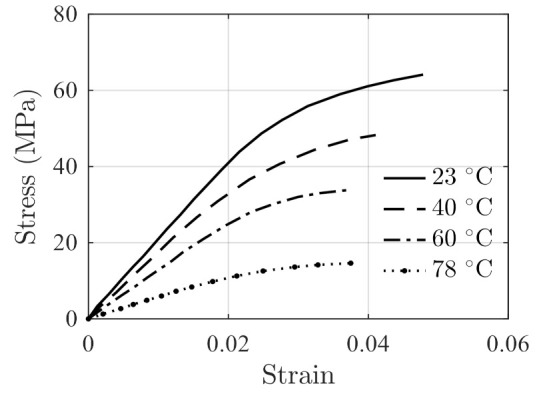
Stress–strain data of FM-73 adhesive at different temperatures reported in Ref. [41].

The performance of the model is further validated using testing data of FM-73 adhesive. The creep data in Ref. [39] and stress relaxation data in Ref. [40] are presented in Figure 7 and Figure 8, respectively. The stress–strain data at different temperatures are obtained from Ref. [41] and are shown in Figure 9. For stress relaxation data in Figure 8, the data at 30 °C, 40 °C, and 60 °C are used for model parameter identification, and the data at 50 °C are used as validation data.

Using the data in Figure 9 and Equations (6) and (7), the strain-hardening rate vs. stress results are extracted and are used to fit the linear strain-hardening equation of Equation (8). The model parameters are identified as

(28) $$\left\{\begin{array}{l} \alpha \left(T\right)=\alpha_{1}\cdot T+\alpha_{2}=-35.94\cdot T+3515\\ \beta \left(T\right)=\beta_{1}\cdot T+\beta_{2}=0.154\cdot T-35.21 \end{array}\right.$$

With Equation (28), the equation of $\Theta \left(T,\sigma \right)$ is written as

(29) $$\Theta \left(T,\sigma \right)=\alpha \left(T\right)+\beta \left(T\right)\cdot \sigma$$

Substituting Equation (29) into the stress relaxation model Equation (18) to have

(30) $$\sigma =\sigma_{0}-\int_{t_{0}}^{t}\left[\alpha \left(T\right)+\beta \left(T\right)\cdot \sigma \right]\cdot \exp\left[\frac{a\left(T,\sigma \right)}{{\left(\tau -t_{0}\right)}^{b\left(T,\sigma \right)}}\right]\cdot H\left[\sigma \right]\cdot H\left[\Theta \left(T,\sigma \right)\right]d\tau ,$$

where $a\left(T,\sigma \right)$ and $b\left(T,\sigma \right)$ are given by Equation (25). Using the stress relaxation testing data at temperatures 30 °C, 40 °C, and 50 °C shown in Figure 8, The parameters in $a\left(T,\sigma \right)$ and $b\left(T,\sigma \right)$ are identified as

(31) $$\left\{\begin{array}{l} a=\left[a_{1},a_{2},a_{3}\right]=\left[0.03810,0.08075,-12.94\right]\\ b=\left[b_{1},b_{2},b_{3}\right]=\left[-7.284\times 10^{-4},-5.632\times 10^{-4},-0.02857\right]. \end{array}\right.$$

**Figure 10:**
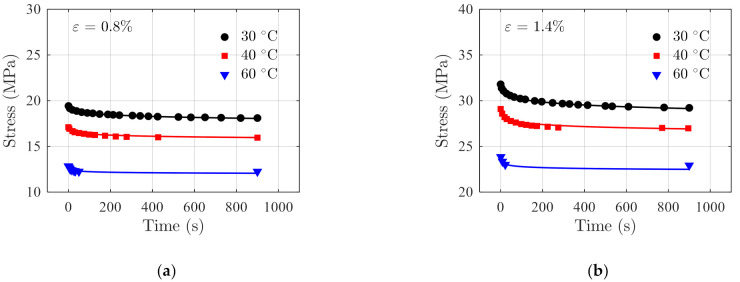
Comparisons of the fitting results (in solid lines) obtained using Equation (30) with the actual stress relaxation data (in discrete markers) in Figure 8 under the constant strains of (**a**) 0.8%, and (**b**) 1.4%.

The fitting results and the actual testing data are compared in Figure 10 where a good agreement between the two is observed under the two constant strains.

**Figure 11:**
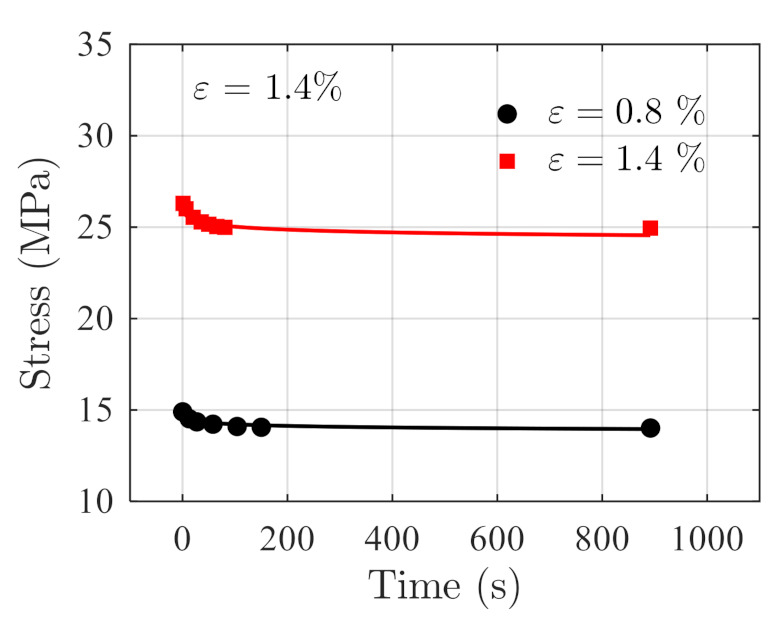
Comparison of the prediction results and the actual stress relaxation data acquired at 50 °C.

The testing data acquired at temperature 50 °C are used as independent validation data. The stress relaxation prediction is made using Equation (30) with T=50 °C. The prediction results and the actual testing data are shown in Figure 11 for comparisons. The RSSs in this case are 0.03689 and 0.2963 under constant strains of 0.8% and 1.4%, respectively, indicating the prediction results are accurate.

### 3.3. Model Comparison

**Figure 12:**
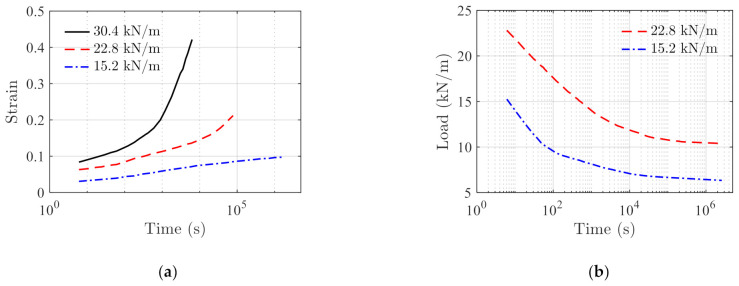
Creep and stress relaxation testing data of HDPE reported in Ref. [38]. (**a**) The creep data, and (**b**) the stress relaxation data.

**Figure 13:**
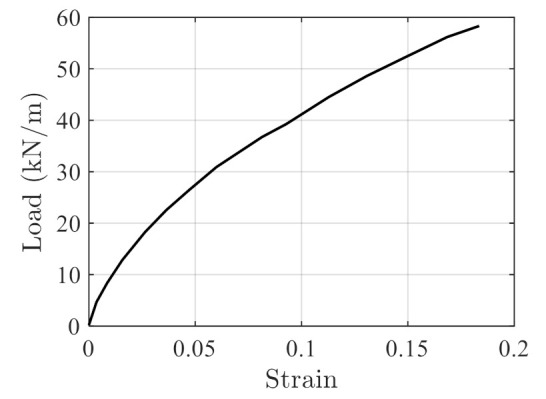
Stress–strain data of HDPE reported in Ref. [37].

To further investigate the performance of the proposed model, the model is compared with three commonly used stress relaxation models in Equation (32)

(32) $$\left\{\begin{array}{ll} S\left(t\right)=\overset{n}{\underset{i=1}{\sum }}S_{i}\cdot \exp\left(-\frac{t}{\tau_{i}}\right), & \mathrm{Parallel}\;\mathrm{Maxwell}\\ S\left(t\right)=S_{\infty }+\overset{n}{\underset{i=1}{\sum }}S_{i}\cdot \exp\left(-\frac{t}{\tau_{i}}\right), & \mathrm{Prony}\;\mathrm{series}\\ S\left(t\right)=S_{0}\cdot \exp\left[-{\left(\frac{t}{\tau }\right)}^{\beta }\right], & \mathrm{KWW}, \end{array}\right.$$

where S is the load, $S_{0}$ is the initial load, t is the time and τ is the material constant. The subscript i is the Maxwell element index and the total number of the Maxwell elements is n. The HDPE testing data for creep and stress relaxation are reported in Ref. [38], and are shown in Figure 12a,b, respectively. In addition, the stress–strain testing data on HDPE are obtained from the Ref. [37], as shown in Figure 13.

The stress–strain data in Figure 13 are transformed into strain-hardening rate vs. stress data using Equations (6) and (7), and the corresponding hardening rate parameters are obtained as a linear rate model of

(33) $$\Theta \left(S\right)=\alpha_{1}\cdot S+\alpha_{2}=-14.10\cdot S+841.5.$$

**Figure 14:**
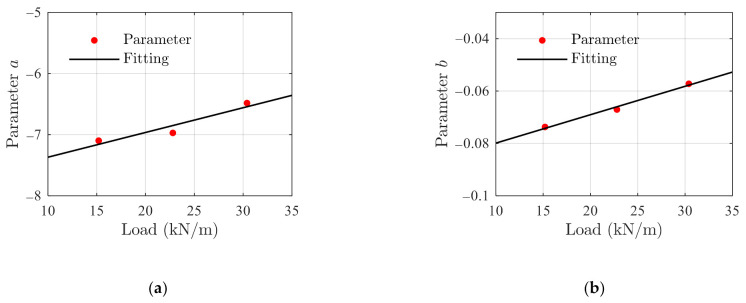
Identified creep parameters and fitting results using Equation (34). (**a**) Parameter a, and (**b**) parameter b.

The fitting creep parameters are obtained using Equation (14) with the data presented in Figure 12a, and the resulting parameters are presented in Figure 14a,b for parameters a and b, respectively. Based on the results, the following linear relationship is established

(34) $$\left\{\begin{array}{c} a=a_{1}\cdot S+a_{2}\\ b=b_{1}\cdot S+b_{2} \end{array}\right..$$

With the above strain-hardening equation and creep parameters, the final stress relaxation model is written as,

(35) $$\sigma =\sigma_{0}-\int_{t_{0}}^{t}\Theta \left(S\right)\cdot \exp\left[\frac{a\left(S\right)}{{\left(\tau -t_{0}\right)}^{b\left(S\right)}}\right]\cdot H\left[S\right]\cdot H\left[\Theta \left(S\right)\right]d\tau .$$

**Figure 15:**
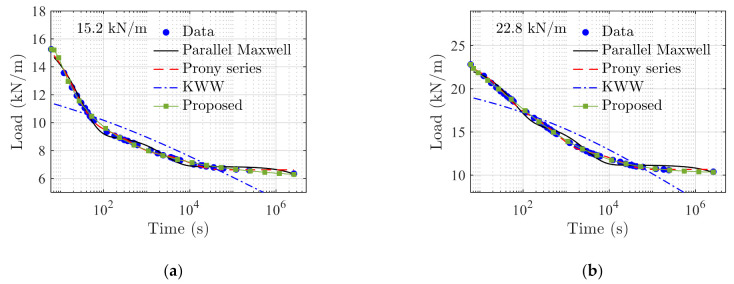
Comparisons of the results obtained using the proposed model and reference models under the two loading cases of (**a**) 15.2 kN/m, and (**b**) 22.8 kN/m.

The same data are used to obtain the model parameters of the parallel Maxwell, Prony series model with three Maxwell elements, and the KWW model, respectively. The model prediction results of those models and testing data are compared in Figure 15. In general, the parallel Maxwell, Prony series, and the proposed method can yield satisfactory fitting results. The KWW model is less accurate as it has only one stretched Maxwell element.

RMSEs and RSSs of the proposed model and reference models under the two loading cases are compared in Table 3, where the proposed model yields the smallest RSS and RMSE. It is worth mentioning that in this case, parallel Maxwell model and Prony series model have six and seven parameters, respectively. The proposed model needs to identify a total number of six model parameters. The number of parameters required to achieve a reliable fitting result in this case are comparable among the three models.

## 4. User Subroutine for Structural Applications

**Figure 16:**
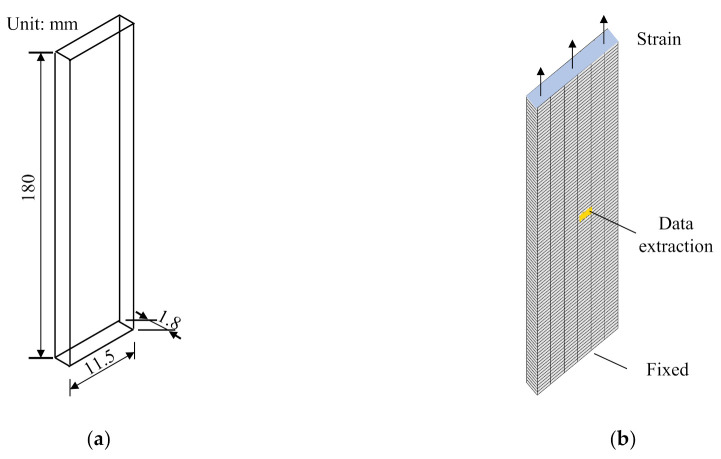
(**a**) The dimensions of the structural specimen, and (**b**) the mesh of structural specimen.

To ease the application of the proposed stress relaxation model to realistic structural problems, a user material subroutine for finite element analysis (FEA) package ANSYS is developed. Taking the FM-73 adhesive in the model validation section as an example, a sheet specimen of 180 × 11.5 × 1.8 mm (Ref. [39]) shown in in Figure 16a is modelled. The specimens are meshed using the quadratic hexahedron elements with an average size of 2 mm, as shown in Figure 16b.

Two loading steps are used to simulate the stress relaxation process of the specimen. First, during the displacement-controlled loading period, the bottom face is fixed in all directions and the initial displacement of the top face is linearly applied from 0 s to 1 × 10^−4^ s. This step imposes the required initial strain to the specimen. Next, the resulting strain is kept constant in the time period of [1 × 10^−^^4^ s, 900 s] to simulate the stress relaxation process of the material. During the stress relaxation step, a total number of 500 uniform sub-steps are prescribed, corresponding to a uniform time increment of dt=1.8 s. An element in the center region of the specimen, as shown in Figure 16b, is chosen for stress data extraction from the FEA results. The extracted stresses at different time instances are used to generate the stress vs. time curves for verification purposes.

**Figure 17:**
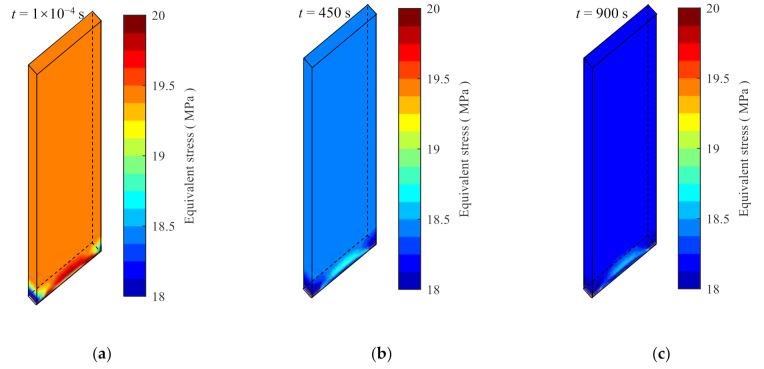
The equivalent stress nephograms calculated by the user material subroutine at 30 °C and 0.8% strain. (**a**) The time is 1 × 10^−4^ s, (**b**) the time is 450 s, and (**c**) the time is 900 s.

**Figure 18:**
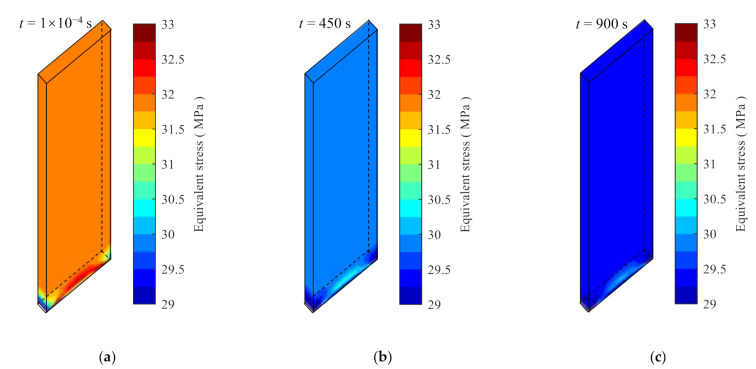
The equivalent stress nephograms calculated by the user material subroutine at 30 °C and 1.4% strain. (**a**) The time is 1 × 10^−4^ s, (**b**) the time is 450 s, and (**c**) the time is 900 s.

The stress relaxation process of the specimen at 30 °C under the constant strains of 0.8% and 1.4% are solved. The results of the equivalent stress contours at three different time instances are shown in Figure 17 and Figure 18 for the strains of 0.8% and 1.4%, respectively. The equivalent stress reduces with time as expected. For the sampled position shown in Figure 16b, the extracted stress from Figure 17c is 18.22 MPa at t=900  s, with a relative error of 0.6409% with respect to the analytical model prediction. For the case of 1.4% strain, the stress at t=900 s is 29.45 MPa with a relative error of 0.8061%.

**Figure 19:**
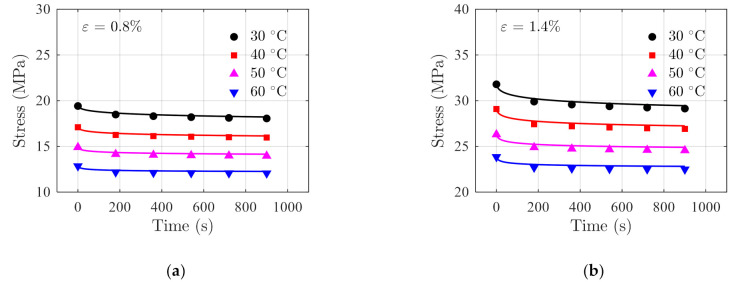
Comparison of the FEA results (in solid lines) and analytical results (in discrete markers) at different temperatures under the constant strains of (**a**) 0.8%, and (**b**) 1.4%.

The stress relaxation for the testing conditions shown in Figure 8 are simulated using the structural model shown in Figure 16b with the developed user material subroutine. The stresses at the sampled position are extracted at all the time instances and are compared with the analytical prediction results, as shown in Figure 19. The results show that the maximum mean relative error, 1.633%, occurs at 60 °C and 0.8% strain, implying that the developed user material subroutine of the proposed model is sufficient for engineering purposes.

## 5. Conclusions

In this study, a general engineering prediction model of stress relaxation for polymer composites was developed. The model is based on the assumption that in the stress relaxation process, the reversible strain is transformed to irreversible strain continuously. A strain-hardening model is employed to incorporate nonlinear elastic deformation of the material, and a creep rate model is used to describe the irreversible strain. The dependence of hardening rate and creep model parameters on temperature was established using the response surface method, allowing for predicting stress relaxation prediction under a range of temperatures. The effectiveness of the developed model was verified using polymer glass data and was further validated using FM-73 adhesive data. Furthermore, the performance of the model was compared with three commonly used stress relaxation models using HDPE data. The user material subroutine of the proposed stress relaxation model was developed and verified for the structural applications.

Compared with the commonly used models such as the parallel Maxwell and Prony series, the developed model has the following two distinct potential advantages. (1) The model eliminates the need for tuning the number of Maxwell elements to reach a required fitting accuracy, and (2) the model takes the nonlinear elastic effect into the stress relaxation process, which can reduce the possible linear approximation error in those commonly used models. Based on the current study, the following conclusions are drawn.

(1) The proposed model provides an alternative to existing stress relaxation models and can account for the nonlinear elastic effect which is absent in the commonly used models based on linear viscoelasticity assumption.
(2) The proposed model can be applied to general polymer composites. In this study, the effectiveness of the model is verified using three different polymer composite materials. Compared with commonly used models, the proposed model yields the smallest statistical error in terms of RMSE in the example case.

It is worth mentioning that the proposed model was established with short-term stress relaxation largely attributed to the physical relaxation of polymer composites. For long-term stress relaxation behaviour due to the combination of physical relaxation and chemical relaxation, the applicability of the proposed model is unknown. In addition, the applicability of the developed to unconfined compression test data is unknown due to the current limited access to such testing data. This aspect will be investigated in the future.

## Figures and Tables

**Table 2:** Results on fitting parameters $\left(a,\;b\right)$ using Equation (14).

T (°C)	σ (MPa)	a	b
400	18	−14.70	−0.02935
450	20.2	−13.22	−0.03352
500	9.5	−12.21	−0.03758
500	14	−11.77	−0.03905
500	20	−11.55	−0.03910
500	25	−11.52	−0.03826

**Table 3:** Comparisons of the performance in terms of RMSE and RSS between the proposed model and reference models.

Index	Load (kN/m)	Proposed	Parallel Maxwell	Prony Series	KWW
RSS	15.2	0.2393	0.9711	0.3827	41.93
	22.8	0.08782	4.126	0.8692	89.76
RMSE	15.2	0.08515	0.1715	0.1077	1.127
	22.8	0.04745	0.3253	0.1493	1.517

## Data Availability

All data are included within the text.
